# Exploring Environmentally Friendly Biopolymer Material Effect on Soil Tensile and Compressive Behavior

**DOI:** 10.3390/ijerph17239032

**Published:** 2020-12-03

**Authors:** Chunhui Chen, Zesen Peng, JiaYu Gu, Yaxiong Peng, Xiaoyang Huang, Li Wu

**Affiliations:** 1 Three Gorges Research Center for Geo-Hazards of Ministry of Education, China University of Geosciences, Wuhan 430074, China; chenchunhui@cug.edu.cn (C.C.); Jia_yuGu@163.com (J.G.); 2 Faculty of Engineering, China University of Geosciences, Wuhan 430074, China; zxc455678125@163.com; 3 Hunan Provincial Key Laboratory of Geotechnical Engineering for Stability Control and Health Monitoring, Hunan University of Science and Technology, Xiangtan 411100, China; 1020172@hnust.edu.cn; 4 Centre for Doctoral Training in Catalysis, Cardiff Catalysis Institute, School of Chemistry, Cardiff University, Park Place, Cardiff CF10 3AT, UK; HuangX17@cardiff.ac.uk

**Keywords:** biopolymer, numerical simulation, micro-behavior, green technology

## Abstract

The study of the high-performance of biopolymers and current eco-friendly have recently emerged. However, the micro-behavior and underlying mechanisms during the test are still unclear. In this study, we conducted experimental and numerical tests in parallel to investigate the impact of different xanthan gum biopolymer contents sand. Then, a numerical simulation of the direct tensile test under different tensile positions was carried out. The micro-characteristics of the biopolymer-treated sand were captured and analyzed by numerical simulations. The results indicate that the biopolymer can substantially increase the uniaxial compressive strength and tensile strength of the soil. The analysis of the microparameters demonstrates the increase in the contact bond parameter values with different biopolymer contents, and stronger bonding strength is provided with a higher biopolymer content from the microscale. The contact force and crack development during the test were visualized in the paper. In addition, a regression model for predicting the direct tensile strength under different tensile positions was established. The numerical simulation results explained the mechanical and fracture behavior of xanthan gum biopolymer stabilized sand under uniaxial compression, which provides a better understanding of the biopolymer strengthening effect.

## 1. Introduction

Traditional cement materials have been employed as stabilizing agents in civil engineering for a long time. Nevertheless, the extensive production and application of these materials have caused serious impacts on the environment, including solid waste, soil contamination, carbon emissions, dust, and water pollution [1,2,3,4,5]. Due to environmental concerned, the exploration of eco-friendly biomixture materials has recently been introduced as a potential replacement of cemented materials. A biopolymer is a high-performance and current eco-friendly material from microorganisms obtained by fermentation. The utilization of biopolymers in civil engineering is a sustainable technology because biopolymers can be used as organic additives in traditional cement materials. According to their composition unit and structure, most biopolymers are polysaccharide polymers. They are high molecular weight polymers formed by the dehydration of multiple monosaccharide molecules. Biopolymers have a profound influence on the soil in terms of the hydroconductivity [6], strength [7], and durability [8] by conducting geotechnical tests [9]. Biopolymers mostly contain hydrophilic groups, and the whole molecule has strong hydrophilicity, which leads to the strong viscosity of aqueous solutions. They can reduce the hydro-conductivity of soil and be used as candidate materials for temporary seepage barriers [10]. The interparticle cohesion provided by the biopolymer reduced soil loss and erosion retain water against evaporation [11]. Meanwhile, the addition of biopolymers can remarkably increase the soil shear strength and compressive strength. Moreover, the use of high content biopolymers can increase the soil strength, which is comparable to concrete to some extent. This strengthening effect of biopolymers is influenced by the biopolymer concentration and type, curing time, dehydration condition, and soil type [12,13,14,15]. Although geotechnical tests have investigated the mechanical behavior of biopolymer-treated soil on a macro scale and explained their possible strengthening mechanism, the interparticle interaction between the soil and failure mechanism during the test remains unknown.

The discrete element method (DEM) is currently a good tool for solving geotechnical problems. The DEM exhibits some advantages in solving discontinuous problems by modeling particle interactions with granular materials, as proposed by Cundall and Strack [16]. The crushable soil/rock materials are assembled by spheres with different contacts. All the particles are rigid bodies while interaction rules are embodied at the particle contact [17]. For discrete sand materials, PFC software can record individual particle displacement, speed, and rotation to analyze the micromovement of sand [18]. With outer static or dynamic loading, deformation occurs at the contact, and PFC can capture the internal micro behavior process of a material. Potyondy and Cundall [19] first proposed the linear parallel bond model to describe the contact of cemented materials. The samples were assembled by rigid spherical particles and jointed by the bond contact model. After applying external forces, the bond contacts become broken and can no longer provide adhesion force [20]. The biopolymer can be regarded as a cemented material that increases soil strength, and the soil particle interaction provided by the biopolymer in particle flow code (PFC) can be quantified by contact model parameters [21]. Considering the computational capacity, as the DEM model often contains thousands of small granular materials, it is appropriate to use PFC to simulate laboratory tests at a small scale. Previous studies used PFC to observe and analyze the specimen micro characteristics with different geotechnical tests. By modeling the direct shear test [22], triaxial test [23], and compressive test [24], the dynamical microbehaviors were monitored and captured to explain the corresponding experimental phenomena in terms of the micro characteristics.

However, biopolymer treated soil has shown improved properties and has been explored at the macro scale. However, the micro-behavior and the in-depth analysis of their characteristics are still unclear. The purpose of this study is to use both experimental and numerical simulations to investigate biopolymer treated soil with respect to macro and microgeotechnical behaviors. We conducted a series of uniaxial compressive tests for soil treated with different biopolymer contents from a macro perspective. For the corresponding numerical simulation, the linear contact bond model was proposed and material parameters were calibrated. Based on these data, direct tensile tests were simulated with different tensile positions which were impossible to carry out in the laboratory. Accordingly, we analyzed the micro parameters, internal force, and crack propagation in detail which were hard to observe via laboratory tests. Finally, the relationship between the tensile strength and compressive strength was analyzed.

## 2. Materials and Methods

### 2.1. Material

**Figure 1:**
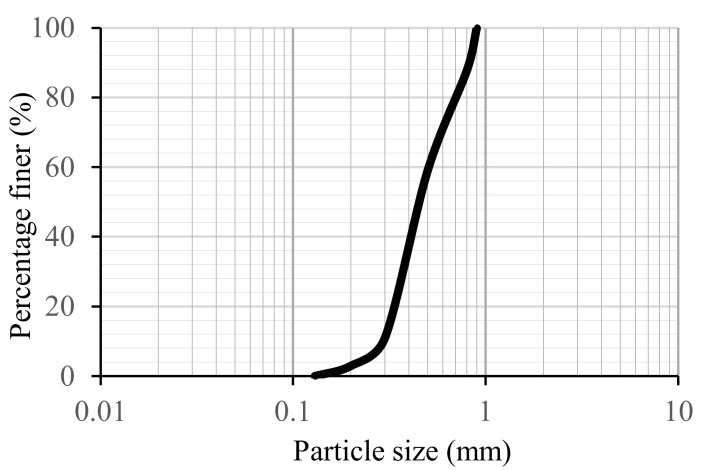
Particle size distribution.

Natural silica sand with a specific gravity of 2.65 was employed for the uniaxial compressive test. The particle size distribution is presented in Figure 1. Commercial microbial exopolysaccharide xanthan gum was used in this research. Xanthan gum is a xanthomonas campestris fermented high molecular weight polysaccharide. Dry xanthan gum is a white powder, that forms a viscous solution when dissolved in water. Under different PH values, temperatures, and ionic strengths, xanthan gum gel still presents stable behavior [25]. It acts as a food thickener, drilling lubrication, and concrete viscosity modifier, and has been used in soil stabilization [26].

### 2.2. Uniaxial Compressive Test

**Figure 2:**
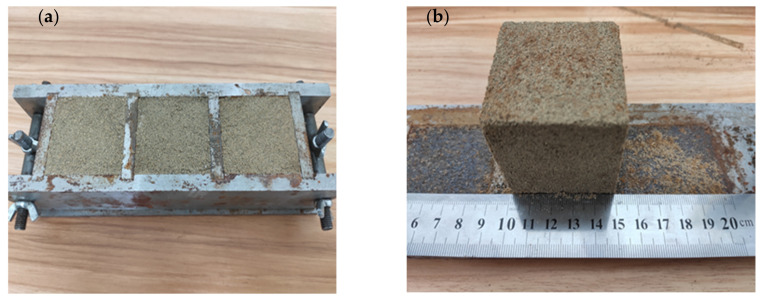
Preparation samples for experimental test ((**a**): sample mold; (**b**): sample dimension).

Due to xanthan gum’s strong hydrophilic property, if poured into the water without proper stirring, the outer sphere will absorb water which then prevents water from permeating into the inter sphere, forming a white clump. Hence, magnetic stirring was used to uniformly mix xanthan gum powder with deionized water. A wide range of biopolymer contents (0.2%, 0.5%, 1%, 1.5% and 2% to the mass of sand) were mixed with 9.6% water, which was the optimum water content of the soil. Then, xanthan gum gel was blended with the sand via blender proper mixing for 10 min and poured into cubic molds with dimensions of 50 mm × 50 mm × 50 mm, as shown in Figure 2. The dry density was set to 1.63 g/cm^3^, and the specimen was compacted layer by layer with the hammer layer by layer. Finally, all the samples were created in triplicate and preserved in a 30 °C oven until a constant weight was reached to represent dry conditions. The uniaxial compressive tests were conducted at 1%/min strain rate. All the tests were conducted under the guidance of Chinese standard GB/T 50123-1999. All tested were carried out in triplicate to minimize experimental error.

### 2.3. Scanning Electron Microscope (SEM)

**Figure 3:**
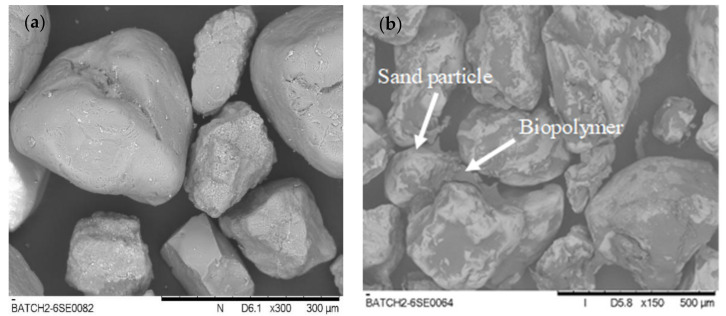
SEM image of biopolymer treated sand and clean sand ((**a**): clean sand with 300 magnification; (**b**): biopolymer treated sand with 150 magnification).

To provide information on the biopolymer and sand interaction for the subsequent numerical simulation model, a scanning electron microscope was adopted. All the SEM images in this study were obtained by TM3030 Tabletop Microscope from Hitachi High-Tech. Samples were loaded on conductive carbon tape (SPI SUPPLIES). The measurement was operated in a vacuum (3–5 Pa) under an acceleration voltage of 15 kV with signals of backscattering electron (BSE). Relevant images and results are displayed in Figure 3.

## 3. Numerical Investigation

**Figure 4:**
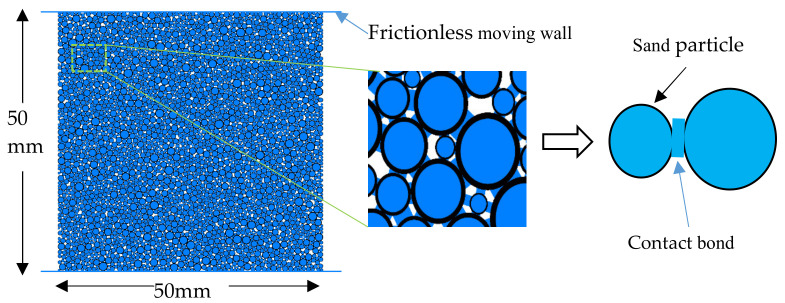
Characteristics of the numerical specimen (all sand particles contacts were linear parallel bond contact which was plot as blue line).

**Figure 5:**
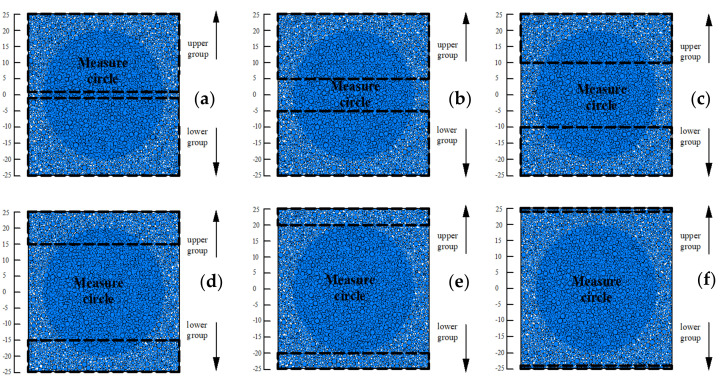
Schematic diagram of specimen tensile movement position ((**a**): tensile position 1~25 mm and −1~−25 mm; (**b**): tensile position 5~25 mm and −5~−25 mm; (**c**): tensile position 10~25 mm and −10~−25 mm; (**d**): tensile position 15~25 mm and −15~−25 mm; (**e**): tensile position 20~25 mm and −20~−25 mm; (**f**): tensile position 24~25 mm and −24~−25 mm;).

The experimental tests provided preliminary outcomes of the geomechanical properties of xanthan gum treated soil. The results indicated that the addition of biopolymer into the soil could largely increase the soil strength which is illustrated in detail in the following sections. To enhance the understanding of biopolymer stabilization soil towards microstructural behavior, particle flow code software was used to analyze the underlying mechanism of the interaction between the treated sands. By simulating the tests, the following processes and problems should be solved and executed:

(1) Contact model. The rigid particles interact with each other at particle surface contacts. Contact modes are assigned at the contacts to develop internal forces for various contact mechanics. The SEM images in Figure 3 present the differences between the clean sand and xanthan gum biopolymer-treated sand. The clean sand has irregular and isolated particles (Figure 3a) while the biopolymer-treated sand is covered and connected by the biopolymer (Figure 3b). The formed hydrogen bonding between the sand particles and xanthan gum provides this bonding connection [27]. In the PFC component, the linear parallel bond exhibits elastic interactions between particles that transmit a normal force, shear force, and moment [17]. When particles are bonded together, they delivered resistance to the particle rotation and therefore exhibit linear elastic to present bond properties. When the bond is broken, it acts as a linear model that cannot resist rotation and tension. Details can be found in the research by Potyondy and Cundall [19]. Thus, the linear parallel bond model can simulate the bonded and unbonded state of the sample particles in the test.
(2) Biopolymer bonding. In the SEM images, biopolymers were distributed in the soil and connected the soil particles. Figure 4 can reproduce this condition by adding a linear parallel contact bond between the sand particles. Contact parameters were embodied in all the particle contacts. Thus, all the particle contacts were set as linear parallel bonds. The different contents of the biopolymer were represented by the different contact parameters.

(3) Sample generation. Previous studies have pointed out that particle sizes and numbers are important factors that affect the simulation time [28]. Considering the computational efficiency and realistic particle size distribution, the particle radius is scaled up 2 times. This is a common way to reduce simulation time with fewer particle balls [29]. The specimen was generated with dimensions of 50 mm × 50 mm in accordance with the experimental sample, as presented in Figure 4. After multiple cycling calculations, the balls reached the equilibrium state in the subsequent test.
(4) Calibrate material behavior. The PFC parameter calibration is a process that reproduces the macro experimental behavior by selecting corresponding numerical parameters to match the experimental data. Due to the complicated interaction in realistic geo-mechanics and simplification in the simulation model, it is difficult to expect these parameters to be precisely recorded and matched with the real conditions. The parameters are given to the PFC components including the ball, wall and contact. As it is difficult to calculate the input parameters through the macroscopic response of the specimen, the most commonly used method of “trial and error” is adapted to calibrate the parameters [30]. Table 1 summarizes the biopolymer binding model parameters.
(5) Remove floaters. When generating ball particles in PFC software, it is evident that there are no contacts around some particles. These floating particles called floaters are isolated and are not connected with the other particles from the initial point of the test. However, with the deformation of the samples during the test, these floaters may contact other particles and influence their properties. Thus, multiple cycles are set to remove the floaters.
(6) Simulating the uniaxial compressive test. Frictionless walls were applied as confined caps at the top and bottom of the sample in the simulation model. The compressive rate was identical to the experimental test. To analyze the micro-behavior of the samples, the stress-strain, internal force, and crack images were tracked and captured during the simulation. The compressive strength was recorded as the average force value on the top and bottom walls during the test.
(7) Simulating the direct tensile test. Unlike the uniaxial compressive test, loading walls were removed in the direct tensile test. A measurement circle was embedded in the specimen to record the axial tensile strength through the built-in FISH language. As the parameters were successfully calibrated to match the experimental test results for the uniaxial compressive test, the direct tensile tests were conducted by using identical parameters to those of the uniaxial compressive test. The particles were divided into an upper group (1~25 mm; 5~25 mm; 10~25 mm; 15~25 mm; 20~25 mm; 24~25 mm;) and a lower group (−1~−25 mm; −5~−25 mm; −10~−25 mm; −15~−25 mm; −20~−25 mm; −24~−25 mm;) according to their position. Then these two groups were given an opposite movement velocity to simulate the direct tensile test. The tensile tests were set for specimens a~f according to their different tensile positions. The schematic diagram is presented in Figure 5.

## 4. Results and Discussion

### 4.1. Comparison of Experimental and Numerical Uniaxial Compressive Test Results

**Figure 6:**
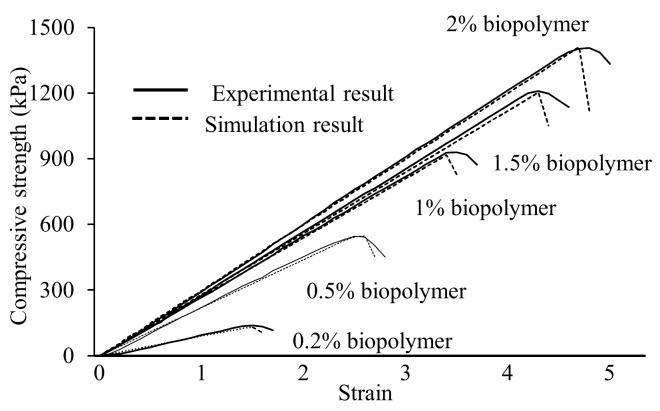
Stress-strain curve of biopolymer treated soil under uniaxial compressive test.

Figure 6 lists the average value of the stress-strain curve of the experimental test results and simulation results of biopolymer treated sand at each content (0.2%, 0.5%, 1%, 1.5%, and 2%). The uniaxial compressive strength of the samples increased with increasing biopolymer content. Compared to cohesionless sand, for which it is difficult to obtain its uniaxial compressive test, there is a similar increasing trend, and the biopolymer has a notable effect on increasing the compressive strength. Simulation results can match the experimental test up to the peak strength. However, after reaching the peak strength, the simulation curves fall off rapidly. This may be caused by the absence of strain localization and the irregularity of the broken fragment. The uniaxial compressive strength of the tested samples increases from 131 kPa (0.2% content) to 1412 kPa (2% content). When the xanthan gum biopolymer is mixed with water, it formed a viscous hydrogel. By properly mixing the xanthan gum biopolymer with sand, these viscous gels are in contact with individual sand particles [31]. There are attachments between the soil particles and biopolymer solution followed by drying to form the adhesive connection [32]. In this case, particles are firmly bonded by the biopolymer to resist outer forces due to increased strength. From the micro parameter perspective, the bond tensile stress and cohesion value of the numerical bond contact in Table 1 improve with increasing biopolymer content, confirming that a higher biopolymer content can directly increase the bonding strength of sand. This eventually leads to an increase in compressive strength. The experimental and numerical results with each xanthan content indicate that the calibrated material behavior and contact model effectively simulate the geotechnical behavior of the samples. It is appropriate to use these numerical parameters to test the specimen tensile strength and analyze the specimen interior micro response and particle behavior.

### 4.2. Tensile Strength

In the SEM image and compressive test simulation, it can be found that the biopolymer provided the most cohesion force to bind the sand particles together. The compression and tension contact force chain development also confirmed the contribution of the biopolymer on the soil cohesion property. Details can be found in the next section. The soil tensile strength directly reflects the mutual attraction of the soil particles which is an important indicator of soil cohesion characteristics. Compared to the compressive strength and shear strength, the soil tensile strength is relatively small and easy to neglect. However, failure modes due to foundation settlement, earth dams and slope are closely related to tensile cracks [33]. Thus, it is necessary to investigate the biopolymer effect on the soil tensile strength of the soil.

To test the tensile strength of the soil, the method can be categorized as the indirect test method and direct test method. The direct test method commonly uses a self-developed apparatus to pull the soil sample and directly test its tensile strength [34]. The main problem with this method is determining how to fix the specimen during the test. In addition, stress concentrations may occur at the specimen fixation position. Indirect tests use other loading and measurement methods to determine the relevant parameters and calculate the soil tensile strength. This is influenced by the specimen size and loading conditions [35]. Only the peak value rather than a series of stress strength data can be obtained by the indirect test. Furthermore, the calculated result cannot precisely match the realistic tensile strength. Both the indirect test and direct test methods cannot observe the influence of the tensile position on tensile strength.

**Figure 7:**
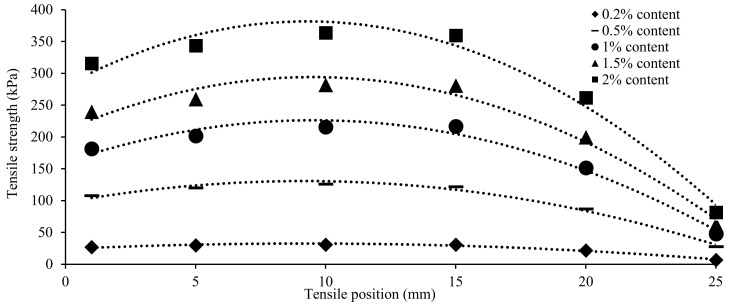
Specimens tensile strength according to tensile position and biopolymer content.

Figure 7 plots different biopolymer treated sand tensile positions and peak tensile strength relationships. The peak tensile strength increased with higher biopolymer content. At each content, the specimen peak tensile strength first increased and then decreased. Thus, the commonly used multiple regression analysis was applied to estimate the tensile strength. The predictive polynomial function and coefficient of determination (R^2^) are listed in equation 1. The second-degree polynomial function is highly correlated (with the high values of R^2^).

**Figure 8:**
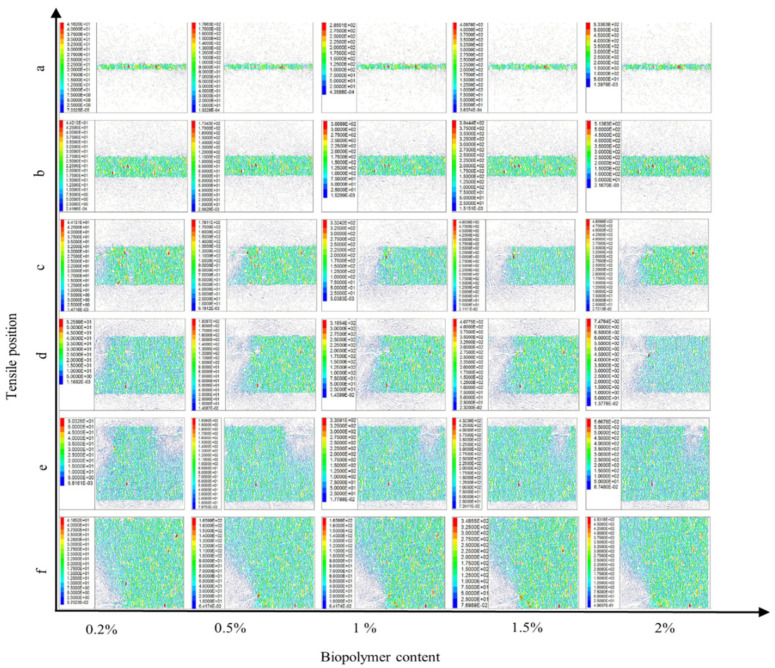
Different biopolymer treated sample force contour under different tensile position.

To analyze the interior force performance, the different biopolymer-treated sample force contours under different tensile positions are presented in Figure 8. The interior forces were illustrated with different colored lines, and the width of the force chain lines was proportional to the force magnitude. From Figure 8, we can conclude that at the same tensile position, the interior force increased with increasing biopolymer content, which can explain the increase in the tensile strength in Figure 7. Chen, Wu and Harbottle [36] found that biopolymer forms a thin film between the sand particles and that this polymer film directly connects the sand particles. This tensile force increased with increasing biopolymer content, which led to an increase in the interior force and tensile strength. When the biopolymer-treated sand was under tension at different positions, there were two force interfaces at each tensile position. The magnitudes of the interior forces were quite different and can be classified as the outer part and inner part according to their relative position to the tensile position. The outer part was located at the moving part of the specimen, where the forces were relatively small. Because all the particles at the outer part were given the same movement velocity, these balls did not have relative displacement. There were no extra interior forces at the moving part to control the deformation. Compared to the outer part, the inner part was located at the extended part of the specimen, which had a high value of forces. The inner part particles did exhibit have movement at first. Due to the contact bond between the particles, inner particles were dragged by the outer part particles from the opposite sides. Thus, the bond contact forces gradually increased to resist tensile deformation.

### 4.3. Internal Force and Crack Propagation Patterns

**Figure 9:**
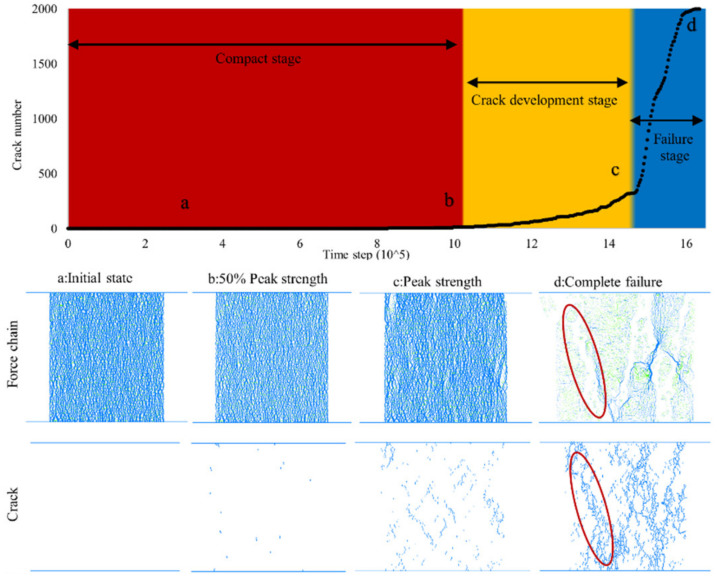
Force chain and crack development of 0.5% biopolymer treated sand under the uniaxial compressive test.

Sand particles are granular materials that convey forces via particle contacts in PFC simulations. During the test, the contact force changes rapidly with the specimen deformation [37]. The discrete element model can effectively visualize these force change networks by plotting force chains. The different contact force chains and fractures vary with the calculation time step. Figure 9 presents the contact force chain and fracture performance of 0.5% biopolymer-treated samples under the uniaxial compression test. The transmitted forces of the sand particles are visualized by green and blue lines: green represents tension forces, while blue represents compression forces. The width of these force chain lines is proportional to the force magnitude. When the specimens experience compressive loading, the aligned particles with internal forces formed the network of the force chain to resist the applied force. Lévy-Véhel [38] mentions that the force chain development is related to strength behavior. According to the development of fracture numbers, the biopolymer-treated sand under uniaxial compression can be described by three phases: the compact stage, crack development stage, and failure stage. At the compact stage (red background in Figure 9), the fracture number remains at zero. Because the contact force chain networks are almost intact, the compressive load is delivered by these networks and no fracture occurs (Figure 9 initial state). The magnitude and form of the internal force maintain the dynamic changes during compression. The slim compression force chain become bold and wide which demonstrates the constant increase in the compressive force provided by the biopolymer at this stage. Chang [39] demonstrated that the grain particle surface coated with xanthan gum biopolymer can enhance interparticle interactions, and the strength of the treated soils depends on the xanthan gum matrix. This cementing effect can be observed in this study and explained in more detail. Under compression, the tension force provided by the biopolymer presents a subhorizontal direction to resist the compressive dilatant deformation. With continuous compression, these forces eventually reach the threshold, they break and can no longer transmit the force which leads to the appearance of cracks. Thus, the sample reaches the crack development stage (yellow background in Figure 9). The overall fracture number increases gradually. The cracks occur (Figure 9b), develop afterward (Figure 9c), and finally penetrate through the sample (Figure 9d). After the compressive strength of the sample reaches its peak strength, the overall fracture number decreases substantially. Crushing is initiated, and relative occurs between the soil particles which results in fragment separation. This phase is called the failure stage (blue background in Figure 9). The break of the contact bonding force is accompanied by the appearance of fractures [40]. This can be confirmed by the obvious failure band marked with circles and concomitant fracture in Figure 9d. The fractures are located in the same position and present a similar shape.

**Figure 10:**
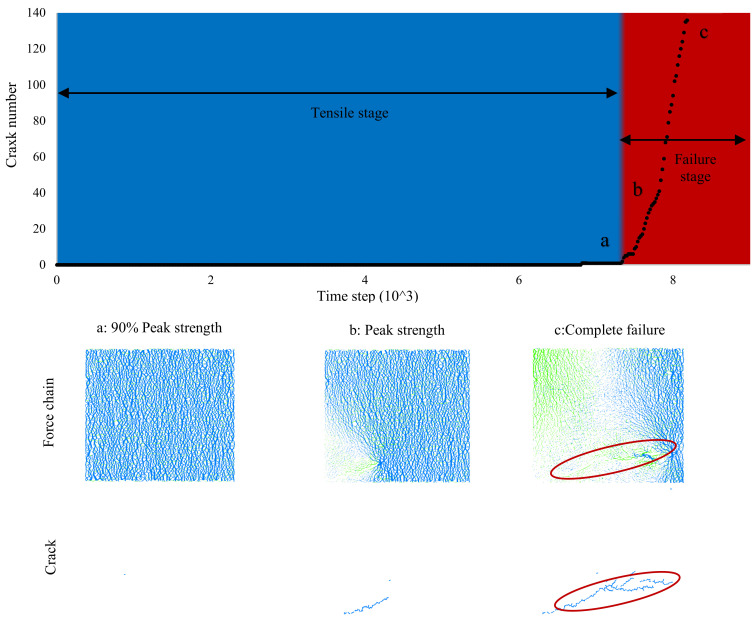
Force chain and crack development of 0.5% biopolymer treated sand under the tensile test.

Figure 10 presents the contact force chain and fracture performance of 0.5% biopolymer treated samples under the tensile test. The tensile test position was selected as the upper group from 24~25 mm and the lower group from −24~−25 mm in Figure 5f. Compared to the uniaxial compression test, the development of the contact force chain and fracture performance of 0.5% biopolymer treated samples under tensile tests exhibited a slightly different performance. The crack number first remained at zero and then increased substantially in Figure 10. It was not until the tensile strength reached 90% of the peak tensile strength that the first fracture appeared. The specimen then started to break from this point and reached failure quickly. Thus, the biopolymer-treated sand under the tensile test could only be described by the tensile stage and failure stage. When the specimen was under compression, cracks started to appear and develop at any possible position. However, when the specimen was under tension, cracks developed along with the first crack position and penetrated through the sample, thus, the crack number of the specimen under tension was much less than that of the specimen under compression. The formation of cracks led to stress redistribution and formed a tensile-zone which eventually resulted in specimen tension failure.

### 4.4. Correlation of Compressive Verse Tensile Strength

Table 2 lists the biopolymer-treated soil tensile strength (qt), uniaxial compressive strength (qu) and the calculation of qt/qu. From the SEM images, it can be seen that the biopolymer occupied the pore space and acted as a bridge to connect sand particles, and biopolymer-treated sand can be regarded as a cemented soil. For cemented soil, the biopolymer content is the main factor influencing the soil strength. Although the biopolymer content had a substantial effect on the compressive strength and tensile strength of the soil, the ratio between the tensile strength and compressive strength can be regarded as a constant value that does not rely on the biopolymer content. Considering that tensile strength varies for different tensile positions, the qt/qu ratio remains a constant and presents a small deviation at each tensile position. This was similar to the results of N.C. Consoli found that the ratio of the lime cemented soil split tensile strength and unconfined compressive strength maintains a unique value of 0.16, independent of other factors [41].

## 5. Conclusions

In this study, both experimental and numerical tests were conducted to investigate the impact of different contents of xanthan gum biopolymers on the uniaxial compression strength and direct tensile strength of sand. The linear contact bond method was proposed to represent the biopolymer binding effect, and the corresponding numerical parameters were calibrated. The micro characteristics of the biopolymer cementation effect were captured and analyzed through the DEM simulation. The following conclusions can be obtained:

(1) The experimental and numerical results with different xanthan contents indicate that the calibrated material behavior and contact model can effectively simulate the geotechnical behavior of the samples. They all indicate that the uniaxial compression strength and tensile strength in the xanthan gum biopolymer-treated sand increase with a higher biopolymer content.
(2) The bond tensile stress and cohesion value in the PFC numerical simulation model increased with increasing biopolymer content, illustrating that a higher biopolymer content can provide a stronger bond. This can also be confirmed by a decrease in the fracture number when the biopolymer content increases.
(3) There was a second-degree polynomial function relationship between the tensile position and tensile strength.
(4) According to the development of the contact force chain and crack propagation pattern in PFC, the behavior from biopolymer-treated sand under uniaxial compression can be classified into three stages: the compact stage, crack development stage, and failure stage, which can be regarded as the tensile stage and failure stage when the specimen is subjected to the tensile test.
(5) Although biopolymer content had a substantial effect on the compressive strength (qu) and tensile strength (qt) of sand, the ratio of qt/qu remained constant at each tensile position, independent of other factors.

## Figures and Tables

**Table 1:** Bond parameters of contact model.

Parameter	Symbol	Biopolymer Content				
		0.2%	0.5%	1%	1.5%	2%
		Value				
Sand particle density (kg/m^3^)	ρ_s_	2600	2600	2600	2600	2600
Bond tensile stress (Pa)	pb_ten	1 × 10^5^	5.8 × 10^5^	6.1 × 10^5^	7.4 × 10^5^	8 × 10^5^
Bond cohesion (Pa)	pb_coh	5 × 10^4^	2 × 10^5^	3.5 × 10^5^	4.6 × 10^5^	6 × 10^5^
Bond normal-to-shear stiffness ratio	kratio	2	2	2	2	2
Friction coefficient	μ	0.34	0.45	0.61	0.64	0.68
Bond effective modulus (Pa)	emod	9.8 × 10^6^	1 × 10^7^	1.3 × 10^7^	1.4 × 10^7^	1.5 × 10^7^

**Table 2:** Biopolymer treated soil tensile strength (qt), uniaxial compressive strength (qu) and the calculation of qt/qu.

Biopolymer Content	qu (kPa)	Tensile Position											
		1~25 mm −1~−25 mm		5~25 mm −5~−25 mm		10~25 mm −10~−25 mm		15~25 mm −15~−25 mm		20~25 mm −20~−25 mm		24~25 mm −24~−25 mm	
		qt (kPa)	qt/qu	qt (kPa)	qt/qu	qt (kPa)	qt/qu	qt (kPa)	qt/qu	qt (kPa)	qt/qu	qt (kPa)	qt/qu
0.2%	131	27	0.206	30	0.229	31	0.237	31	0.237	22	0.168	7	0.053
0.5%	548	108	0.197	120	0.219	126	0.230	122	0.223	87	0.159	28	0.051
1%	920	182	0.198	202	0.220	216	0.235	217	0.236	152	0.165	48	0.052
1.5%	1204	240	0.199	260	0.216	282	0.234	281	0.233	200	0.166	63	0.052
2%	1412	316	0.224	344	0.244	364	0.258	360	0.255	262	0.186	82	0.058
Average value	-	-	0.205	-	0.225	-	0.239	-	0.237	-	0.169	-	0.053
Standard deviation	-	-	0.0100	-	0.0101	-	0.0098	-	0.0104	-	0.0090	-	0.0024
